# The “DrownSafe” Project: Assessing the Feasibility of a Puppet Show in Teaching Drowning Prevention to Children and Parents

**DOI:** 10.3390/children11010019

**Published:** 2023-12-23

**Authors:** Lucía Peixoto-Pino, Roberto Barcala-Furelos, Begoña Paz-García, Cristina Varela-Casal, Miguel Lorenzo-Martínez, Adrián Gómez-Silva, Javier Rico-Díaz, Antonio Rodríguez-Núñez

**Affiliations:** 1 Facultade de Ciencias da Educación, Universidade de Santiago de Compostela, 15706 A Coruña, Spain; lucia.peixoto@usc.es (L.P.-P.); javier.rico.diaz@usc.es (J.R.-D.); 2 CLINURSID Research Group, Psychiatry, Radiology, Public Health, Nursing and Medicine Department, Universidade de Santiago de Compostela, 15706 A Coruña, Spain; antonio.rodriguez.nunez@usc.es; 3 REMOSS Research Group, Facultade de Ciencias da Educación e do Deporte, Universidade de Vigo, 36005 Pontevedra, Spain; marpaz@uvigo.es (B.P.-G.); cristina.varela.casal@uvigo.es (C.V.-C.); miguel.lorenzo.martinez@uvigo.es (M.L.-M.); adrian.gomez.silva@uvigo.es (A.G.-S.); 4 ESCULCA Knowledge and Educational Action Research Group, Universidade de Santiago de Compostela, 15706 A Coruña, Spain; 5 Faculty of Nursing, Universidade de Santiago de Compostela, 15782 A Coruña, Spain; 6 Paediatric Critical, Intermediate and Palliative Care Section, Hospital Clínico Universitario de Santiago de Compostela, 15706 A Coruña, Spain; 7 Collaborative Research Network Orientated to Health Results (RICORS): Primary Care Interventions to Prevent Maternal and Child Chronic Diseases of Perinatal and Developmental Origin, Instituto de Salud Carlos III, 28029 Madrid, Spain; 8 Simulation and Intensive Care Unit of Santiago (SICRUS) Research Group, Health Research Institute of Santiago, University Hospital of Santiago de Compostela (CHUS), 15706 A Coruña, Spain

**Keywords:** drowning prevention, learning, training, schoolchildren, parents, puppets show, lifeguard, low-cost intervention, basic life support

## Abstract

Drowning remains a prominent global pediatric health concern, necessitating preventive measures such as educational initiatives for children and caregivers. In this study, we aimed to assess the feasibility and educational effectiveness of an interactive puppet show centered on teaching water safety to children and parents. A 30 min original theater performance, featuring two actors and three puppets (a girl, a crab, and a lifeguard), was conducted. Subsequently, 185 children (aged 4 to 8) and their 160 parents (134 mothers and 26 fathers) participated in this quasi-experimental study. Pre- and post-show tests were administered to evaluate knowledge and behaviors regarding aquatic environments. Prior to the puppet show, 78% of the children exhibited basic aquatic competency. Only 33% considered swimming alone risky. Following the intervention, 81.6% of the children changed their perception of the risks of solo beach activities, showing improved knowledge regarding contacting an emergency number (from 63.2% to 98.9%, *p* < 0.001). The intervention increased parents’ intention to visit lifeguard-patrolled beaches and improved their CPR knowledge with regard to drowning victims by 58.8%. In conclusion, a drowning prevention puppet show positively impacted children and parents, potentially enhancing safety behaviors during water-related leisure activities, warranting its consideration part of comprehensive drowning prevention strategies.

## 1. Introduction

Childhood drowning is a concerning public health problem that affects communities worldwide [1,2], being the third leading cause of global child mortality among children aged 5 and older [3]. Incidents involving children in aquatic environments have multifactorial causes, including lack of aquatic competence, absence of direct supervision, and caregivers’ negligence [4,5,6].

Education must play a crucial role in drowning prevention, and different school programs, from preschool to secondary education, have demonstrated benefits related to drowning prevention [7,8,9,10,11]. Childhood drowning incidents are multifactorial, so the approach to their prevention should also involve parents or caregivers, as they reinforce learning and safe behaviors in the aquatic environment [12,13,14,15]. However, there is a gap in the knowledge regarding which activities or educational approaches could be effective alternatives for younger children as well as regarding how to address them from a family perspective. Furthermore, there is general agreement that community intervention has a beneficial effect on preventing drowning [16].

A primary purpose of drowning research should be to provide low-cost, wide-ranging, and easily replicable educational resources and strategies to mitigate cultural or economic gaps, particularly focusing on low-income countries where children are more vulnerable [3]. In the quest for effective strategies, scientific evidence has shown that theatrical performances, especially those involving puppets, have great potential for teaching content related to injury prevention and health education due to their incorporation of fantasy elements and imagination, allowing recipients to actively engage in the teaching–learning process [17]. 

The use of puppets has a long tradition in Early Childhood Education and Care (ECEC) [18]. A recent review of puppet use for children aged 1 to 9 identified up to four theoretical perspectives (constructivism, psychology, sociology, and arts) that support the constructs of communication, fantasy, storytelling, and friendship among young children [19]. However, the use of puppets in ECEC has been declining in recent years [19]. Nevertheless, evidence has shown how interactive simulation with puppets can be particularly effective in children’s healthcare [20]. The pedagogical use of puppets is supported by theories that emphasize the importance of imaginative experiences in learning [19], encouraging the fantastic transition from children’s imagination to key elements in early scientific research and science education [21]. Puppets offer a unique and effective educational approach, providing opportunities for imaginative play, communication, and engagement that may not be as easily achieved through traditional methodologies, especially in the field of health, where content can elicit rejection or phobias. Puppets can overcome these barriers and facilitate effective communication and expression among young children [18]. In the healthcare field, puppets have been used to detect strokes or seek medical assistance, demonstrating their potential in intervention programs and health education [22]. In the field of water safety, to the best of the authors’ knowledge, no research has been conducted on drowning prevention using puppets.

Therefore, based on the hypothesis that a puppet show, specifically written with messages geared towards safety in aquatic environments, would improve the knowledge and attitudes of both children and their parents regarding drowning prevention and the activation of the chain of survival, we aimed to assess the feasibility and immediate effects of this innovative teaching strategy and tool.

## 2. Materials and Methods

A quasi-experimental study was designed, comprising three phases: creation of the puppet show, recruitment of the sample, and pre- and post-intervention evaluation. The research team consisted of 8 experts, including 1 pediatrician, 1 puppeteer, 2 lifeguards, 2 university professors specializing in the arts, and 2 university professors who were experts in drowning prevention. This research was approved by the Ethics Committee of the Faculty of Education and Sports Sciences of the University of Vigo under the code 06-170123 and was conducted in accordance with the ethical principles of the Helsinki Convention. 

### 2.1. Creation of the Puppet Show

The play was an adaptation of the children’s book “The rat who wanted to learn to swim” (“O rato con as que quería aprender a nadar”) [23]. This book pedagogically addresses the most frequent incidents reported in scientific literature to be known causes of child drowning [4,5,10,24] and covers the following topics: the meaning of safety flags on beaches, how to activate emergency services in case of witnessing a drowning, and basic tips for safe bathing (such as the importance of never bathing alone, even when using floating devices). The group of experts adapted the book’s messages and scripted the puppet play. This play depicted the aquatic adventures of the protagonist puppet, designed in the likeness of an approximately five-year-old girl. Throughout the performance, this puppet experienced two drowning incidents: one at the beach and another in a pool. The plot of the play is summarized below:

“A girl (the puppet) was at the beach and decided to swim in the sea without adult supervision, despite the warning of the red flag. This led her to aspirate water and cough (non-fatal drowning—grade 1). Subsequently, the same girl re-entered the water equipped with a ring-shaped float. Due to the wind, she was carried farther out to sea, requiring a rescue by the lifeguard and the incident only resulted in a warning (water rescue). After this incident, upon returning home, she attempted to retrieve a ball from her pool but slipped and fell, initiating the drowning process. Her mother promptly called the emergency number 112, and a lifeguard (another character) performed cardio-pulmonary resuscitation (CPR), explaining the steps to the public. The protagonist puppet was revived in the scene (non-fatal drowning—grade 6). The story concludes with a moral emphasizing prevention, the importance of respecting sea warning flags, always bathing under adult supervision, remembering the emergency number 112, and the steps of CPR”.

**Figure 1:**
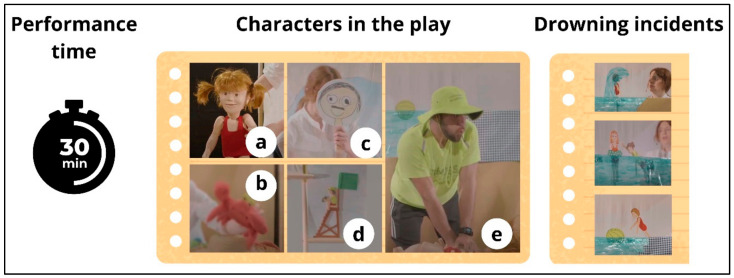
Performance time, characters, and drowning incidents in the show. Legend: (**a**) main character, the puppet Lis; (**b**) puppet crab; (**c**) the mother, an actress; (**d**) the puppet lifeguard; (**e**) the real lifeguard, an actor.

Therefore, the cast of the play (Figure 1) included (a) the main character, a 5-year-old girl named Lis (puppet) who served as the protagonist around whom the story revolves; (b) a crab (puppet), who was her friend and reinforced the educational messages; (c) the mother (actress), who alternated between offering educational advice and displaying confusion and a lack of attention during drowning; (d) a lifeguard (puppet), who performed the sea rescue; and (e) another lifeguard (actor), who taught the drowning CPR protocol (5 rescue ventilations followed by a sequence of 30 chest compressions and 2 ventilations) and saved the main character (Lis). The recording of the performance is available online via the following link: https://www.youtube.com/watch?v=1Z3gI_dgb9Q, accessed on 19 December 2023.

### 2.2. Sample

A total of 345 subjects participated in this study, comprising 185 children (85 boys and 100 girls) aged between 4 and 8 years (mean age: 6.2 ± 1.1 years) and 160 parents (134 mothers and 26 fathers) aged between 29 and 56, with an average age of 41.7 ± 4.8 years. The study involved 11 theatrical performances conducted in different cities and towns in Galicia (northwest Spain). Each performance accommodated between 20 and 40 people and was advertised in the local press and on the institutional website of the University of Vigo. The inclusion criteria for the child participants encompassed an age range of 4 to 8 years of age, parental authorization, and voluntary participation. For adults, inclusion required a parental (father or mother) relationship with the child attending the puppet show. Participants did not receive any form of compensation for their involvement, and attendance at the puppet show was free. The children’s legal guardians provided informed consent for the use of their data in this research.

### 2.3. Intervention, Variables, and Evaluation

**Figure 2:**
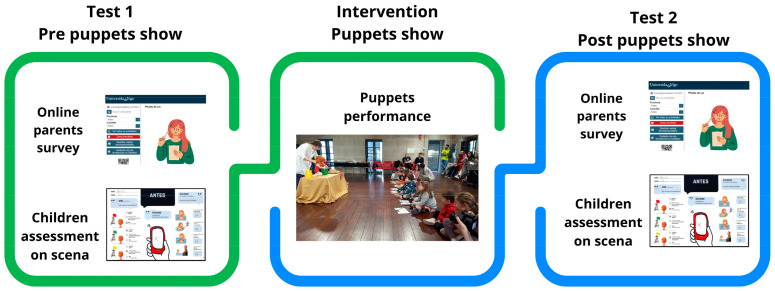
Intervention design and evaluation.

The puppet show was scheduled to last 30 min and was consistently performed by the same actress and characters, as well as under optimal conditions of light, sound, and space. The research design focused on two aspects: (a) the pre- and post-intervention knowledge of the children and (b) the attitudes and knowledge regarding drowning prevention among the parents (Figure 2).

#### 2.3.1. Children’s Knowledge of Drowning Prevention (Pre- and Post-Intervention)

**Figure 3:**
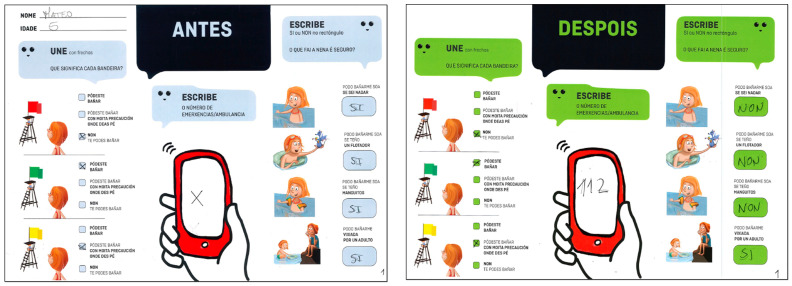
Design of the children’s evaluation tool for drowning prevention.

The evaluation tool was designed in the format of a children’s school card, wherein children were required to answer a series of questions presented in the form of illustrations (Figure 3). This evaluation system was employed based on a previous pilot study that demonstrated its methodological feasibility for similar age groups [10]. The design and iconography of the evaluation sheet were developed by two professionals in graphic design and arts, with extensive experience in the illustration and creation of children’s materials.

Before the puppet show, the children individually filled out the evaluation form (Test 1). During the 30 min period following the performance, they, once again, individually completed the reverse side of the evaluation form, which displayed the same set of questions (Test 2).

The variables were grouped into three blocks: (1) the association between flag colors and their corresponding meanings, (2) knowledge of the emergency number, and (3) safe bathing behaviors (being alone, using a float, wearing sleeves, and/or being supervised by an adult). For evaluation, a dichotomous scale of correctness or error was used for each item, alongside a cumulative variable reflecting the total number of correctly answered items.

#### 2.3.2. Parents’ Behaviors and Knowledge Regarding Drowning Prevention

For the parents, the research team designed a questionnaire consisting of 10 questions. Following a discussion process based on the focus group technique, 3 questions were eliminated, resulting in a consensus-derived total of 7 questions. Questions Q1 and Q2 were administered before the puppet show to assess the children’s aquatic skills according to the classification of Szpilman et al. [4] and determine whether the parents’ exhibited any significant distractions or lapses in attention during their child’s bath at any point. Questions Q3–Q6 were presented both before and after the performance. The purpose of these questions was to assess whether the puppet show had influenced parents’ preferences regarding the choice of beaches with the presence of lifeguards, exploring the use of flotation devices as a measure for drowning prevention, investigating comprehension of the meanings of sea state flags, and analyzing knowledge of CPR. Finally, Q7 was intended to determine if the puppet show induced any changes in knowledge regarding drowning prevention (Table 1). Parents completed this survey electronically through an email invitation sent during the registration process for the puppet show and during the week following their attendance at the event.

### 2.4. Statistical Analyses

All analyses were conducted using the statistical package IBM SPSS for Windows (version 25.0. Armonk, NY, USA: IBM Corp). The descriptive results for qualitative variables are presented as absolute and relative (%) frequencies of responses, while means and standard deviations (SD) are provided for continuous variables. The McNemar test was used to analyze the differences in the children’s responses before and after the intervention. The differences between the number of correct responses provided by the children before and after the intervention were analyzed using the Wilcoxon test. For all analyses, the significance value was set at *p* < 0.05.

## 3. Results

### 3.1. Children’s Knowledge of Drowning Prevention

Table 2 shows the differences in the children’s knowledge regarding water safety before and after the intervention. Overall, the children answered an average of 5.5 ± 1.5 out of 8 questions correctly before the intervention, whereas after the intervention, the number of correct answers increased to 7.6 ± 0.9. Thus, there was a significant improvement in the children’s overall knowledge of water safety following the intervention (Wilcoxon *Z* = 10.746; *p* < 0.001). More specifically, the percentage of correct responses significantly increased (*p* < 0.05) for all items for which the children were questioned, except for bathing under adult supervision, where there was little room for improvement as a high number of correct responses had already been obtained in the initial test. In the third block, there was an approximate 50% increase in the percentage of correct responses for the variables bathing alone, using a flotation device, and using armbands.

### 3.2. Parents’ Behaviors and Knowledge Regarding Drowning Prevention

Table 3 displays the information on parental behaviors and knowledge with respect to water safety before and after the intervention. Around 80% of the children lacked the ability to swim or had only basic flotation skills. Moreover, 17.5% of the parents acknowledged that they had inadvertently left their children unsupervised near water or allowed them to bathe without supervision at some point.

The majority of parents (42.5%) indicated that they did not consider the presence of lifeguards to be a determining factor when selecting a beach or pool, while 21.9% stated that they did not take it into account at all. In contrast, 21.3% expressed a preference for beaches with lifeguards, and 14.4% considered it a crucial factor. However, there was a significant shift in the perception of lifeguards’ importance following the intervention. Most parents (31.3%) stated a preference for beaches with lifeguards, and 27.5% considered their presence a determining factor. Regarding the use of armbands and floaters as a preventive measure against drowning, the majority of parents reported that their children used them sometimes (29.4%) or always (29.4%), while others indicated that they never (25.0%) or almost never (16.3%) used such devices. After the intervention, parents expressed a greater inclination toward their children using these devices. Specifically, 28.8% of parents stated their children would use them sometimes, and 40.0% indicated they would use them always.

With respect to the knowledge of the meaning of the three flags denoting sea conditions (green, yellow, and red), before the intervention, 91.9% of the parents were familiar with their meaning. After the intervention, all parents reported being aware of their meanings. Furthermore, there was a significant increase in the percentage of parents who considered themselves knowledgeable with regard to performing pediatric CPR, rising from 27.5% to 86.3% following the intervention.

Overall, although most parents (42.5%) already perceived themselves to be highly aware of the behaviors and recommendations presented in the performance, others reported a slight (13.8%), moderate (26.3%), or substantial (17.5%) increase in their understanding of child drowning prevention. Furthermore, the parents’ overall evaluation of the theatrical performance averaged 3.8 ± 0.4 on a 4-point Likert scale.

## 4. Discussion

This study was designed to analyze the knowledge and perceptions of children and their parents regarding aquatic safety. It was also intended to promote a more proactive awareness of drowning prevention through attendance at a puppet show. The main findings are as follows: (a) The children demonstrated a high level of knowledge about the meaning of flags but had limited awareness of the relevant emergency number. A high percentage of children believed they could bathe without adult supervision. Some adults acknowledged failures in supervision while their children were in the water. (b) The parents’ choice of aquatic spaces did not correspond to safety criteria, such as the presence of a lifeguard, even though the majority of children possessed only basic aquatic competence or did not know how to swim. (c) After the puppet show, the children exhibited increased confidence in their preference for bathing supervised by adults. Among the parents, half of them considered that the performance would lead to changes (to a greater or lesser extent) amounting to safer and more preventive behaviors in aquatic environments while also enhancing their knowledge of CPR.

In the battle against drowning, the World Health Organization (WHO) advocates for community-based educational initiatives that focus on enhancing public awareness and education regarding the use of aquatic spaces and training children and bystanders in safe bathing, safe rescue procedures, and CPR [3]. Accessibility is also promoted, so the creation of educational materials and resources must be a priority strategy. In addition, various previous efforts using comics and stories [10] have proven effective in enhancing children’s understanding of water safety. In this regard, puppet plays have been implemented in diverse health areas for educational purposes [25,26]. However, to the best of our knowledge, their effect on preventing drowning has never been studied.

The use of puppets in the shape of children serves a pedagogical purpose since they can be perceived as peers by other children. This educational approach is practical and cost-effective, and it also allows for addressing false beliefs within an imaginary scenario [27]. However, children’s knowledge gained from puppets may not necessarily align with their knowledge gained from real-world social agents, such as adults [28]. To mitigate potential discrepancies between the imaginary and the real, a collective activity involving parents and children was promoted. In this activity, puppet representations were used to impart new knowledge and encourage safer behaviors related to drowning prevention at the family level.

In the first phase, the primary aim was to identify the baseline. It became apparent that the children participating in this study had basic aquatic competence, as reported by their parents. Aquatic competence is defined as the set of skills essential for surviving common drowning situations and even includes the ability to identify a swimmer in distress, call for help, or perform a safe rescue [29]. However, young children often lack developed aquatic skills, consequently heightening their vulnerability in water [3]. Moreover, the current findings revealed that a significant proportion of the children did not recognize bathing alone as a potentially dangerous behavior, and approximately 20% of the parents admitted to instances of providing inadequate supervision during their children’s aquatic activities, even on more than one occasion. This situation is not coincidental, as other studies have also identified that between 15% and 30% of caregivers have left young children unsupervised for periods ranging from 1 to 5 min during bathing [5]. 

The puppet show emphasized this key concept, highlighting unsupervised access to water as the primary trigger for drowning among young children [4,5,30,31]. Throughout the storyline, the puppet experienced two non-fatal drownings, both of which could have been prevented if parents had been present. While some may believe that children carrying floating devices or knowing how to swim might relax their attention, it is crucial to note that knowing how to swim does not render them “drown-proof” [12]. The American Academy of Pediatrics emphasizes that parents and caregivers should never—even for a moment—leave children alone or in the care of another child in bathtubs, swimming pools, or open water [4]. The primary preventive strategy is supervision [5,11], defined as direct, hands-on supervision, where adults are within arm’s reach of a child [4]. Adequate supervision comprises three key components: proximity, attention, and continuity [32]. By following this approach, parents play an active role and become aware of the importance of supervision. After the intervention, half of the parents indicated that their knowledge about preventing drowning increased after attending the puppet show. Moreover, over 80% of the children stated that bathing alone was an incorrect behavior. Another positive outcome from the puppet show was an increase in parents’ intention to visit supervised beaches, recognizing that lifeguards provide additional security [4,33].

In the puppet show, the recognition of sea state flags was promoted, as it is directly relates to drowning prevention. Prior to the intervention, the parents and children already possessed a high level of knowledge regarding their meanings. Following attendance at the puppet show, flag recognition reached nearly 100%. These findings suggest that using simple visual elements is an effective strategy. However, there is still no universal consensus regarding this symbology. In various regions worldwide, such as Spain, the flags represent traffic light colors, but in other areas, up to six or seven flags coexist, differing in color and even shape, potentially hindering comprehension, especially among children. This raises the question of why there is an almost unanimous global consensus on most danger symbols or road signs, while the same does not hold true for symbols and signs used in aquatic environments.

In the drowning survival chain, the first step is prevention, while the second is recognizing aquatic distress and asking for help [34]. Therefore, this puppet show was also intended to teach the audience how to ask for help. Contacting emergency services not only activates the chain but also serves as a preventive measure against further rescue attempts by laypeople, which can potentially result in the drowning of both the victim and the rescuer [35,36]. Overall, over half of the children in this study were already aware of the European emergency telephone number (112). However, after the intervention, nearly 100% of them indicated they would know who to call in the event of an emergency occurring in an aquatic setting.

The final step in the chain of survival for drowning is to provide necessary care [34]. Our puppet show addressed this aspect by incorporating recommended adaptations for managing cardiac arrest resulting from drowning (rescue ventilations and 30 chest compressions plus two ventilations). Tobin et al. [37] observed neurologically favorable survival rates among children who received bystander compressions and ventilations. Therefore, it is imperative to train parents in the application of conventional CPR and encourage its use within this demographic. The aim of this intervention was to move from the standard recommendation for laypeople of “just compress” towards the recommendation of “compress and ventilate”, specifically in cases involving children and/or individuals experiencing cardiorespiratory arrest due to drowning. In the theatrical performance, an actor (lifeguard) successfully resuscitated the puppet (the main protagonist), and during the CPR demonstration, the actor interacted with the audience, explaining key guidelines according to the European Resuscitation Council’s recommendations for specific circumstances (drowning) [38]. Upon completion of the intervention, 86% of the parents reported knowledge of the CPR techniques indicated for drowning. This dissemination led to the majority of the adults being theoretically aware of the peculiarities of performing cardiopulmonary resuscitation in cases of asphyxia.

### 4.1. Practical Implications

Young children represent a particularly vulnerable group due to their limited ability to assess risks effectively and insufficiently developed swimming skills, which impede their autonomy in aquatic environments [16]. Preventing aquatic incidents requires a multifaceted approach, with education playing a pivotal role. Evidence has shown that educational activities involving children, parents, or communities have a positive impact on drowning prevention. The challenge, however, lies in providing cost-effective interventions (for greater accessibility) that are pedagogically efficient and replicable. An example of such an intervention is the “Kim na escola” project promoted by the Brazilian Aquatic Rescue Society (SOBRASA) [39], in which, through an interactive show involving puppets, lifeguards, and children, emphasis is placed on drowning prevention. Puppets can serve as an alternative satisfying all these criteria and can be integrated into various programs implemented in different countries, both in live performances and in online versions (YouTube, Instagram, and TikTok).

### 4.2. Limitations of This Study

This research has some limitations that must be pointed out. This study was confined to a specific Spanish region with a strong connection to the sea; hence, it is plausible that different answers might have been observed in other locations or among individuals with distinct cultural profiles. This puppet show may have an impact on locations with conditions similar to those in this study. Not all places around the world have children drowning in the same spaces and under the same circumstances. Puppets can serve as an educational medium, but adaptation to different communities and differences in circumstances leading to drowning is necessary.

An important limitation is the absence of a control group with which to unequivocally attribute the acquired knowledge to the intervention (the puppet show). Some initial responses revealed elevated values (e.g., the significance of sea state flags), which could be explained by the attending families having a pre-existing interest in water safety. This could introduce a recruitment bias. Additionally, there was a bias that was challenging to control concerning certain responses, as a correct answer does not necessarily correlate with correct behavior. This research did not assess the retention of learning over the long term. Future studies should aim to evaluate the retention of learning and its relationship with drowning prevention in real-life situations.

Therefore, future research should aim to investigate the effects of parental and child behaviors on actual aquatic incidents.

## 5. Conclusions

A community educational model based on a puppet show is effective in promoting knowledge and safer behavioral practices for the prevention of drowning, targeting both young children and their parents. The use of puppets can be an engaging, interactive method for enhancing connections with both children and adults, effectively conveying essential messages that contribute to reducing the incidence of drowning. Through the puppet-based approach, parents can shift their mindsets towards adopting safer and more proactive behaviors in drowning prevention while gaining new knowledge. Hence, encouraging and facilitating parental participation in educational activities tailored for children is highly recommended.

## Figures and Tables

**Table 1:** Questionnaire for parents distributed before and after the puppet show.

Information Pre-Intervention	Comparison between Pre- and Post-Intervention	Information Post-Intervention
Q1. What aquatic competence does my child have? 1. My child does not have swimming or flotation skills. 2. My child has basic floating skills. 3. My child is able to swim using more than one stroke and has advanced floating skills. 4. My child is able to swim in all 4 strokes. Crawl, backstroke, breaststroke, and butterfly. 5. My child is a swimmer with risk analysis and rescue knowledge.	Q3. With family, we go to beaches or pools with lifeguard supervision. 1. It is not a criterion that we consider when choosing a beach. 2. Somewhat agree. If you have a lifeguard, better, but it is not a selection criterion. 3. Strongly agree. Preferably we look for supervised beaches. 4. Totally agree. It is one of the beach selection criteria.	Q7. Did the puppet show “Drown-Safe” lead to any kind of change in your idea of drowning prevention? 1. It did not entail any change. I was already very aware. I respected and knew all the behaviors and recommendations shown in the work. 2. It improved a bit. Only in some aspects or behaviors not too relevant. 3. It improved somewhat. Now I feel that I will be more aware when my children are in the water. 4. It improved a lot. I am going to change my behaviors towards preventing the drowning of my children.
	Q4. My child uses armbands and floatation devices as a preventative measure against drowning. 1. Never 2. Almost never 3. Sometimes 4. Always	
Q2. Have you ever been distracted while your child was near water or bathing alone without supervision? 1. Yes, but it is not an oversight, my child already bathes or swims alone. 2. Yes, more than once. 3. Yes, once. 4. Never.	Q5. As a parent, do you know the meaning of the three state sea flags (green, yellow, and red)? 1. I have doubts about the meaning of the three. 2. I have doubts about the meaning of two. 3. I have doubts about the meaning of one 4. Yes, about the three.	
	Q6. Do you know how to perform CPR adapted for a drowning victim? 1. No 2. Yes	

**Table 2:** Differences in children’s knowledge of water safety before and after the intervention (*n* and % of correct responses).

		Before	After	McNemar χ^2^ (*p*-Value)
Block 1. Knowledge of flag colors	Red Flag	173 (93.5%)	184 (99.5%)	7.692 (0.003)
	Yellow Flag	174 (94.1%)	183 (98.9%)	7.111 (0.004)
	Green Flag	161 (87.0%)	184 (99.5%)	21.043 (<0.001)
Block 2. Emergency number	Number: 112	117 (63.2%)	183 (98.9%)	64.015 (<0.001)
Block 3. Safe bathing behaviors	Bathing alone	61 (33.0%)	151 (81.6%)	80.827 (<0.001)
	Using a flotation device	78 (42.2%)	170 (91.9%)	86.260 (<0.001)
	Using armbands	73 (39.5%)	163 (88.1%)	82.510 (<0.001)
	Adult supervision	177 (95.7%)	180 (97.3%)	0.364 (0.549)

**Table 3:** Differences in parents’ behaviors and knowledge regarding water safety before and after the intervention (*n* and % of correct responses).

		Before	After
Q1. What aquatic competence does my child have?	1. My child does not have swimming or flotation skills.	32 (20.0%)	
	2. My child has basic floating skills.	94 (58.8%)	
	3. My child is able to swim using more than one stroke and has advanced floating skills.	32 (20.0%)	
	4. My child is able to swim in all 4 strokes: crawl, backstroke, breaststroke, and butterfly.	1 (0.6%)	
	5. My child is a swimmer with risk analysis and rescue knowledge.	1 (0.6%)	
Q2. Have you ever been distracted while your child was near water or bathing alone without supervision?	1. Yes, but it was not an oversight; my child already bathes or swims alone.	3 (1.9%)	
	2. Yes, more than once.	5 (3.1%)	
	3. Yes, once.	20 (12.5%)	
	4. Never.	132 (82.5%)	
Q3. With family, we go to beaches or pools with lifeguard supervision.	1. It is not a criterion that we consider when choosing a beach.	35 (21.9%)	3 (3.8%)
	2. Somewhat agree. If you have a lifeguard, that is better, but it is not a selection criterion.	68 (42.5%)	30 (37.5%)
	3. Strongly agree. Preferably, we look for supervised beaches.	34 (21.3%)	25 (31.3%)
	4. Totally agree. It is one of the beach selection criteria.	23 (14.4%)	22 (27.5%)
Q4. My child uses armbands and floatation devices as a preventative measure against drowning.	1. Never	40 (25.0%)	21 (26.3%)
	2. Almost never	26, 16.3%)	4 (5.0%)
	3. Sometimes	47 (29.4%)	23 (28.8%)
	4. Always	47 (29.4%)	32 (40.0%)
Q5. As a parent, do you know the meaning of the three state sea flags (green, yellow, and red)?	1. I have doubts about the meaning of the three.	4 (2.5%)	0 (0.0%)
	2. I have doubts about the meaning of two.	2 (2.0%)	0 (0.0%)
	3. I have doubts about the meaning of one	7 (4.4%)	0 (0.0%)
	4. Yes, about the three.	147 (91.9%)	80 (100%)
Q6. Do you know how to perform CPR adapted for a drowning victim?	1. No	116 (72.5%)	11 (13.8%)
	2. Yes	44 (27.5%)	69 (86.3%)
Q7. Did the puppet show “Drown-Safe” lead to any kind of change in your idea of drowning prevention?”	1. It did not entail any change. I was already very aware. I respected and knew all the behaviors and recommendations shown in the work.		34 (42.5%)
	2. It improved a bit. Only in some aspects or behaviors not too relevant.		11 (13.8%)
	3. It improved somewhat. Now I feel that I will be more aware when my children are in the water.		21 (26.3%)
	4. It improved a lot. I am going to change my behaviors towards preventing the drowning of my children.		14 (17.5%)

## Data Availability

The data presented in this study are available on request from the corresponding author. The data are not publicly available due to containing information protected by the Spanish Organic Law on Personal Data Protection and Digital Rights Guarantee.
